# Laboratory Profiles of Treatment-Seeking Subjects With Concurrent Dependence on Cannabis and Other Substances: A Comparative Study

**DOI:** 10.5812/ijhrba.10993

**Published:** 2013-12-23

**Authors:** Rizwana Quraishi, Raka Jain, Biswadip Chatterjee, Arpita Verma

**Affiliations:** 1 National Drug Dependence Treatment Centre, Department of Psychiatry, All India Institute of Medical Sciences, New Delhi, India; 2 Department of Psychiatry, All India Institute of Medical Sciences, New Delhi, India

**Keywords:** Cannabis, Urine, Liver Function Tests, Leukocyte Count

## Abstract

**Background:**

Cannabis is one of the most widely used illicit drugs in India and worldwide. It is considered to have a minimal effect on physical health.

**Objectives:**

The aim of this study was to compare the laboratory profiles of treatment-seeking patients who were cannabis dependent, and drug users who concurrently use other substances, with non-users.

**Materials and Methods:**

Medical records of patients, whose urine was tested for the detection of cannabis within the last year, were considered for the study. The inclusion criteria for the study group were; co-morbid diagnosis of cannabis dependence according to DSM-IV TR criteria, positive urine drug screen for cannabis, and at least one biochemical or hematological examination report during the treatment period. The subjects who underwent all of the above mentioned tests, but who were negative for any psychoactive substance with no past or current history of substance use, were placed in the control group.

**Results:**

A total of 51 subjects fulfilled the inclusion criteria for the study group and 30 subjects were considered as controls. There was no significant difference found between the demographic profiles of the subject and control groups. The mean duration of cannabis use in the patients was 9.53 ± 8.06 years. Serum levels of; bilirubin, SGOT (serum glutamic oxaloacetic transaminase), SGPT (serum glutamic pyruvic transaminase), total protein, alkaline phosphatase, ESR, and eosinophil counts, were raised in; 13.7%, 15.6%, 33.3%, 17.6%, 37.2%, 75% and 5.8% of subjects, respectively. The relative monocyte count was lower than normal in 92% of cases. Physical complaints were reported in 98% of subjects. The two groups showed significant differences in serum alkaline phosphatase [t (79) = 6.5, P ≤ 0.01], TLC [t (79) = 2.36, P = 0.03] and hemoglobin levels [t (79) = 5.50, P ≤ 0.01].

**Conclusions:**

Abnormal laboratory parameters were observed in patients with cannabis dependence. The study emphasizes the need for regular physical examinations and laboratory investigations for cannabis users.

## 1. Background

Throughout the world, cannabis (*Cannabis sativa*) is used for diverse purposes which range from medicinal use, and recreation, to severe forms of dependency. In India, cannabis has both religious and social sanctions, and the plant is widely available in uncultivated areas, which has made it a common drug of abuse in the country. Bhang (wet/dry paste of the leaves), ganja (powdered flowering stem of the plant), and charas (resin extracted from the plant), are some of the most common forms of cannabis used in India. While it is estimated that the global prevalence of cannabis use in the general population is estimated to be 2.8-4.5% (1), the prevalence in India is about 3% among the general population, and 11% among the treatment-seeking population (2).

The psychological side-effects of cannabis are well-known. Symptoms of cannabis intoxication include; mild euphoria, relaxation, perceptual alterations, and feeling intensification, to; anxiety, panic attacks, and psychotic symptoms (3). Chronic cannabis use is associated with psychosis and schizophrenia (4). Prolonged use also results in sustained deficits in cognitive functions such as; verbal learning, memory and attention (5), which in turn is associated with poor educational attainment and high rates of unemployment (6).

The physical harm caused by cannabis is less well-known. In adults, chronic bronchitis, lung cancer, myocardial infarction, hepatotoxicity, decreased sperm count and motility, gynaecomastia in males, suppression of ovulation among females, low birth weight and delayed visual system development among the newborns of cannabis using females, have been reported (7-11). Some previous studies have reported effects on the blood including; increases in hemoglobin concentrations, packed cell volume, and red blood cell counts, and these have been attributed to chronic exposure to smoke and carbon monoxide similar to that among tobacco smokers (12). Studies have observed the different effects of cannabinoids on peripheral leukocyte counts, while some studies have showed an increase in eosinophil counts and peripheral blood lymphocytes, others have reported a significant decrease in the same conditions (13-16). These studies suggest that contrary to common belief, chronic cannabis use does have adverse effects on physical health. Moreover, most cannabis users referred to specialized de-addiction centers are co-dependent on other substances like; nicotine, alcohol, and opioids, which can further increase the adverse effects of cannabis.

## 2. Objectives

To compare the laboratory profiles of treatment-seeking concurrent cannabis users, and other substance dependent subjects, with psychoactive substance non-users, visiting a tertiary care center in northern India.

## 3. Materials and Methods

The study was conducted at the National Drug Dependence Treatment Centre, All India Institute of Medical Sciences, New Delhi, which is a government-funded research and treatment center. It draws patients from the entire northern and eastern regions of India. As part of the evaluation process; a detailed socio-demographic profile, substance use profile, psychiatric and medical history, were recorded in a semi-structured form during the first treatment contact. The psychiatric and medical examinations were carried out by a psychiatrist and a medical officer, respectively. Subsequently, routine urine screening, using thin layer chromatography (TLC), or a Cassette test (Alfa Diagnostics, USA), for the presence of various substances such as; cannabis, various opioids, amphetamines, and some benzodiazepines, were conducted in order to confirm a history of substance use. Other laboratory investigations were done per request. These included biochemical parameters such as; total bilirubin, serum glutamic oxaloacetic transaminase (SGOT), serum glutamic pyruvic transaminase (SGPT), total protein, albumin, and alkaline phosphatase levels, using standard reagents (Roche Diagnostics) on an Echo plus biochemistry autoanalyzer (Logotech Pvt. Ltd, India). Hematological parameters including; hemoglobin, total and differential leukocyte counts, red blood cell counts, and erythrocyte sedimentation rates (ESR), were measured.

For the current study, records of all patients whose urine was screened for cannabis within the previous 12 months were retrieved and evaluated to diagnose cannabis dependency according to DSM-IV TR criteria (17). Inclusion criteria for the study were; documented urine screen positive for cannabis on at least one occasion during the treatment period, and at least one documented biochemical and hematological investigation during the same period. In addition, data was also collected from subjects who had similar socio-demographic profiles to the substance using subjects and who had undergone biochemical and hematological investigations as previously mentioned, but without any history of drug use or any previous history of medical illness. A total of 51 substance using subjects and 30 controls fulfilled the study criteria.

The study was conducted in accordance with the ethical standards of the Declaration of Helsinki (1975), and complete confidentiality was ensured. Descriptive statistics were used for the analysis. An independent t-test was used to compare the parametric data, and a Mann-Whitney U test for the non-parametric data. The data was analyzed using STATA data analysis and statistical software (ver. 8.0).

## 4. Results

A total of 51 substance using subjects and 30 controls fulfilled the study criteria.

### 4.1. Socio-demographic Profile

All subjects were male, and the mean age of the cannabis using subjects was 30.12 ± 10.23 years, while the mean age of the control group was 30.83 ± 9.53 years, and no significant difference was found between these groups, t (79) = 0.31, P = 0.76. The majority of the subjects 90.2% (n = 46) were younger than 25 years-of-age. In both groups, more than 95% of the subjects were literate, while 82.4% (n = 42) of the cannabis users had a lower socio-economic status. All of the subjects in the control group were employed, while 52.9% (n = 27) of the cannabis users were unemployed.

### 4.2. Cannabis and Other Substance Use

The cannabis and other substance user profiles are shown in Table 1. The duration of cannabis use varied from ten months to forty years, and 37% (n = 19) of the subjects had been using it for more than ten years. Ganja was the most common form of cannabis used. All subjects were co-dependent on nicotine and 64.7% (n = 33) of the subjects were also opioid dependent. Physical and psychological complications were observed in 95% (n = 48) of the subjects, but only 35.3% (n = 18) were aware of the health risks associated with cannabis use. Physical complaints such as; weakness, malaise, and weight loss, were reported by all subjects, while specific complaints including breathing difficulties, were reported by 7.8% of the subjects (n = 4). Specific psychological complications reported were: auditory hallucinations (n = 4, 7.8%), persistent behavioral disturbances such as; aggressiveness and violent anger outbursts (n = 3, 5.8%), and sustained sadness and suicidal ideation (one subject). The control group reported no significant physical or psychological complaints. 

**Table 1.** Cannabis and Other Substance User Profiles

Variables	
**Age of initiation Into substance use, mean ± SD, y**	15.31 ± 4.7
Below 15, No. (%)	30 (58.9%)
15-24, No. (%)	17 (33.3)
25-44, No. (%)	4 (7.8%)
**Duration of substance use, mean ± SD, y**	14.8 ± 9.6
**Duration of cannabis use, mean ± SD, y**	9.53 ± 8.06
**Type of cannabis, No. (%)**	
Bhang	8 (15.7)
Ganja	34 (66.7)
Charas	9 (17.6)
**Co-morbid substance use (with cannabis dependence), No. (%)**	
Nicotine dependence	51 (100)
Alcohol dependence	15 (29.6)
Opioid dependence	33 (64.7)
Intravenous opioid use	11 (21.6)
Benzodiazepine dependence	5 (9.8)
Other	2 (3.9)

### 4.3. Laboratory Profile

The biochemical and hematological profiles are presented in Table 2. Serum bilirubin, protein, SGOT, and alkaline phosphatase levels, were elevated in 13.72%, 15.6%, 33.3% and 37% of the substance-using subjects, respectively. Out of the 17 substance-using subjects with abnormal SGOT, 47% (n = 8) were opioid dependent, 41.17% (n = 7) were alcohol dependent, and 11.7% (n = 2) were injecting drug users. Out of the 19 subjects with deranged alkaline phosphatase, 84.2% (n = 16) were opioid dependent, and 36.84% (n = 7) were alcohol dependent. The hematological profiles of the substance user group showed raised ESR counts in 75% (n = 38) of the subjects. Relative monocyte counts were below the normal range in 92% (n = 47) of the subjects. 

**Table 2.** Laboratory Profiles

Variables	Control, Mean ± SD, (n = 30)	Cannabis Dependent	
		Mean ± SD, (n = 51)	No. (%), With Abnormal Values
**Urea**	25.60 ± 60	27.41 ± 7.58	1 (2)
**Serum creatinine**	0.82 ± 0.61	0.91 ± 0.23	1 (2)
**Total bilirubin**	0.81 ± 0.21	0.8 ± 0.62	7 (13.72)
**Total protein**	7.76 ± 0.75	7.26 ± 0.77	8 (15.68)
**Albumin**	4.62 ± 0.42	4.22 ± 0.87	4 (7.84)
**SGOT^a^**	34.75 ± 11.8	37.53 ± 18.5	17 (33.33)
**SGPT^a^**	37.8 ± 18.8	29.77 ± 17.0	9 (17.64)
**Alkaline phosphatase**	98.82 ± 26.46	217.53 ± 95.84^b^	19 (37.25)
**Hemoglobin**	13.26 ± 0.96	11.52 ± 1.52^b^	51 (100)
**ESR^a^**	20.83 ± 10.14	26.51 ± 14.04	31/41 (75.61)
**TLC^a^**	6 934.48 ± 1699.00	7 737 ± 1 302.76^b^	1 (2)
**DLC^a,c^**	-	-	-
**Neutrophils**	61.18 ± 7.06	65.0 ± 7.78	4 (7.5)
**Lymphocytes**	34.27 ± 7.06	29.06 ± 4.39	1 (2)
**Eosinophils**	1.36 ± 1.12	4.00 ± 5.01	3 (5.88)
**Monocytes**	2.45 ± 2.60	2.14 ± 1.59	47 (92)

^a^ Abbreviations: DLC, dimensional liquid chromatography; ESR, erythrocyte sedimentation rate; SGPT, serum glutamic pyruvic transaminase; SGOT, serum glutamic oxaloacetic transaminase; TLC, thin layer chromatography.

^b^ Significant at 0.05 (independent sample t-test).

^c^ Data missing for 15 subjects in the control group.

Mean levels of serum alkaline phosphatase [t (79) = 6.5, P ≤ 0.01], and TLC [t (79) = 2.36, P = 0.03], were significantly high, and the level of hemoglobin was significantly low [t (79) = 5.50, P ≤ 0.01], in the substance users compared with the control group.

Among the substance users, the injecting drug users (IDUs), when compared to non-IDUs, had significantly increased neutrophil counts [t (79) = 1.89, P = 0.04], and serum albumin levels [t (79) = 1.84, P = 0.05]. The laboratory parameters did not show any significant differences between the subjects with and without other comorbid substance uses (alcohol or opioid).

## 5. Discussion

Studies on substance users are often difficult to conduct due to the ‘hidden’ nature of their population. Their profiles differ widely from the general population; however, insights into their characteristics can be gained by studying treatment seekers. Most of the studies on cannabis have emphasized the psychological aspects, not the physical harm, as it is often considered to lack major physical complications (18). This study shows the laboratory profiles of cannabis dependent treatment seeking subjects from India who were co-dependent on other substances, and to compare them with substance non-users. It is difficult to recruit subjects of cannabis dependence without any associated complications or comorbid substance use since they often do not seek treatment because of social and cultural reasons. The subjects in this study were young and educated, but their rate of unemployment was significantly high; indicating that occupational dysfunction may lead to substance abuse. More than half of the subjects started substance use before the age of 15 years.

A large proportion of the subjects showed abnormal liver related parameters (especially serum alkaline phosphatase) which supports previous studies that have reported hepatomegaly and abnormalities in liver enzymes caused by cannabis in the absence of jaundice (12). Although alcohol and opioids are also known to affect liver function (19), in this study, no significant difference in the values of biochemical parameters were noted between; alcohol or opioid users and non-users. Hematological parameters such as hemoglobin were significantly lower than in non-users. This is in contrast with most of the previous studies which have reported an increase caused by functional hypoxia (13). The high prevalence of raised ESR indicates the frequency of infection among this population. Significant increases in neutrophil counts were found among IDUs, which is in accordance to earlier reports from the center on IDU users (20). Eosinophil counts were found to be high in 6% of cases, and neutrophil count abnormalities were found in 7.5% of subjects, which support previous studies on cannabis users (14). The lymphocyte counts showed a normal level in contrast to previous studies, which reported low lymphocyte counts in humans (13). A low mean relative monocyte count (2.14 ± 1.59) was observed, which was also reported by Mukhtar et al. (15).

Some of the limitations of this study were as follows: a small sample size, co-dependence on other substances, and a retrospective method of data collection, which restricts its generalization to the community setting. Some of the findings, such as anemia and liver function derangement, could have been caused by other associated factors like; poor nutrition, occult blood loss, or chronic infections such as; tuberculosis, hepatitis and sexually transmitted diseases, which are common. However, this study adds to the limited number of studies on chronic cannabis users from the northern part of India and provides some preliminary information for future prospective studies.
